# Human cell types important for Hepatitis C Virus replication *in vivo *and *in vitro*. Old assertions and current evidence

**DOI:** 10.1186/1743-422X-8-346

**Published:** 2011-07-11

**Authors:** Dennis Revie, Syed Zaki Salahuddin

**Affiliations:** 1 Department of Biology, California Lutheran University, 60 W. Olsen Rd., Thousand Oaks, California, 91360 USA; 2 Basic Research, California Institute of Molecular Medicine, 1879 Portola Rd., Unit J, Ventura, California, 93003 USA

## Abstract

Hepatitis C Virus (HCV) is a single stranded RNA virus which produces negative strand RNA as a replicative intermediate. We analyzed 75 RT-PCR studies that tested for negative strand HCV RNA in liver and other human tissues. 85% of the studies that investigated extrahepatic replication of HCV found one or more samples positive for replicative RNA. Studies using *in situ *hybridization, immunofluorescence, immunohistochemistry, and quasispecies analysis also demonstrated the presence of replicating HCV in various extrahepatic human tissues, and provide evidence that HCV replicates in macrophages, B cells, T cells, and other extrahepatic tissues. We also analyzed both short term and long term *in vitro *systems used to culture HCV. These systems vary in their purposes and methods, but long term culturing of HCV in B cells, T cells, and other cell types has been used to analyze replication. It is therefore now possible to study HIV-HCV co-infections and HCV replication *in vitro*.

## Introduction

The majority of the current research on human hepatitis C virus (HCV) is unusual in that it does not study the natural virus, but instead studies are based on synthetic constructs and their manipulations. This article will limit itself to the analysis of viral RNA and proteins from patient sera or on replicating virus isolated from cells infected with HCV from patient sera. HCV is an old entity, but the bulk of our knowledge is fairly recent. Human hepatitis B virus (HBV) was identified in the 1960s. The hepatitis cases that could not be identified either as Hepatitis A or Hepatitis B were referred to as non-A non-B hepatitis. HCV was molecularly cloned and identified in the late 1980s [1] and an almost complete sequence was obtained soon thereafter [2]. HCV is a single stranded, positive sense RNA virus that is variable in size, but approximately 50 to 80 nm in diameter. It is a member of *Hepacivirus *group in the family *Flaviviridae.*

**Figure 1:**
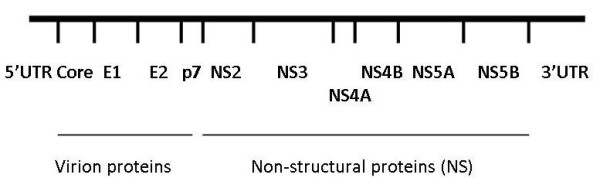
**Genome organization of HCV**.

The HCV genome is about 9.6 kb in length. One large precursor protein is synthesized from an open reading frame of over 9024 nucleotides. This polyprotein is then cleaved to produce 10 proteins (Figure 1). These include three structural proteins at the amino end of the RNA transcript: Core and two envelope proteins (E1 and E2). An ion channel protein p7 is formed by cleavage of E2 [3]. Next are six proteins that are not in the viral particle (NS2, NS3, NS4A, NS4B, NS5A, and NS5B). In addition, a protein called F or ARFP can be produced from a frame-shift of the Core protein [4]. The viral RNA contains a 5' untranslated region (5'UTR), typically 341 nucleotides, that is highly conserved between the virus strains [5]. This region contains an internal ribosome entry site (IRES) for translation. The 3' end of the virus contains an untranslated region (3'UTR) that is 200 to 235 nucleotides long. It contains, in order, a variable region, a poly U/UC stretch, and a highly conserved 98 nucleotide sequence [6].

It is now accepted that HCV is a cause of liver diseases and a number of other lymphoproliferative disorders such as mixed cryoglobulinemia (MC) [7-10] and Non-Hodgkin's lymphoma (NHL) [11-14]. Other lymphoproliferative disorders may also be associated with HCV infection [15]. Although the pathogenic process is not well understood, HCV infection progresses slowly and often ends in chronic diseases. Over years, chronic infection may end in fibrosis and cirrhosis. A large percentage of patients develop liver failure or other complications of cirrhosis such as hepatocellular carcinoma.

Classical thinking was that HCV only infects hepatocytes and hence the inflammation of liver. Many studies of HCV have investigated infection of other cell types such as peripheral blood mononuclear cells (PBMC). In fact, evidence has begun to accumulate that liver cells are only one of the targets of HCV infection. The previous idea was established due to the identifiable disease associated with liver. However, viral replication has been reported in B cells, T cells, monocytes, macrophages, and other macrophage-like cells such as Kupffer cells and dendrocytes. The study of HCV has been dominated for many years by molecular approaches directed towards understanding protein functions that may lead to therapy or vaccines. Yet the biology of HCV has unfortunately been largely neglected.

We are reviewing reports of the biology of HCV, as this area of HCV research is in major need of understanding. In particular, studies investigating the *in vivo *host range of HCV as well as *in vitro *culture systems that can be used to study the biology of this virus will be discussed.

## Sites of HCV replication *in vivo*

For the *in vivo *studies outlined below, we have looked at papers that analyzed the presence of HCV RNA. HCV is a positive strand RNA virus that replicates through a negative strand intermediate. Papers that detected or measured the presence of the negative strand of HCV by a variety of methods were analyzed, as were papers that described methods to detect the non-structural (NS) proteins of HCV, since they are not found in the virion. If the abstract did not mention negative strands of HCV or NS proteins, we probably did not include the study in our analysis, except for the papers that looked at quasispecies or binding of the virus.

## HCV can bind and enter extrahepatic cells

The information below summarizes a number of studies that represents binding of selected viral proteins but they do not address the question of viral entry. One of the first studies on the subject of HCV in serum was on virus concentrated by ultracentrifugation, followed by testing the concentrate for positive and negative strands [16]. These were tests done on cell free virus, and RNase and detergent sensitivity were tested. Positive strands were resistant to both RNase and detergents, while negative strands were resistant to either RNase or detergent. The investigators concluded that positive strands were probably protected in an enveloped core, while negative strands were membrane protected but not in a protein core.

For replication to occur, HCV must bind and enter cells like any other virus. Although there has been extensive study of the receptors that HCV uses to enter cells, almost all of these have been *in vitro *studies using model systems [17]. One HCV binding study investigated the types of HCV in blood [18]. They found that about 1% of HCV was in cells and 4 to 5% of the virus was not cell bound and existed in immune complexes. The binding of free virus to Molt-4 (T cells) and U937 (monocytes) cell lines could be saturated, but binding to platelets could not. The binding to the cell lines was inhibited by anti-HCV IgG, with the inhibition exhibiting different kinetics for Molt-4, U937, and platelets. This suggests that HCV binds to different cell types using a combination of receptors. Immune complexes that contain HCV bound Molt-4 and U937 to lesser extents, but platelets bound free virus or virus particles in complexes in equal proportions.

HCV replication can be studied by examining particular steps in the replication process of the virus. One study analyzed binding of a soluble version of HCV E2 (sE2) to cell types separated from PBMC, including monocytes, monocyte-derived dendritic cells (mDC), plasmacytoid dendritic cells (pDC), natural killer cells (NK), B cells, CD4^+ ^T cells, and CD8^+ ^T cells [19]. sE2 binding largely paralleled CD81 expression. However, monocytes, DCs, and B cells also had CD81 independent binding. They also found variability in binding efficiency from different HCV sE2 variants. Another study used flow cytometry and antibodies against Core and E1 to study binding and entry into peripheral blood leukocytes (PBL) [20]. They found that after 1 hour, staining was found on the surface of the cells, but after 24 hours, there was no surface staining and 28% of the cells were intracellularly stained. Negative strand HCV RNA could be seen at 24 hours post-infection (PI). A study of the attachment of HCV to Daudi cells (B cells) used RT-PCR to measure HCV bound or inside cells [21]. They found that immunocomplexed HCV did not attach as well as the free virus. The attachment was best at pH 7.0, and HCV then probably entered cells after 90 minutes post-infection.

Since much of the HCV in serum is complexed with antibodies, one study investigated infection of monocytic (U937 and Monomac-6) and T lymphocytic (MOLT-4 and Jurkat) cell lines [22]. They stimulated these cell lines with phorbal myristate (PMA) and interferon-γ to stimulate Fc receptor expression before infecting with immune complexes of HCV. Entry was measured by RT-PCR. Non-stimulated cells showed no viral entry, while HCV negative strand was detected for up to 7 days after infection in monocytic cells. Little or no binding was seen for the lymphocytic cell lines. In the monocytic cell lines, monoclonal antibodies to FcγRII, a low affinity immunoglobulin G receptor, abolished binding by immune complexes, suggesting that HCV can enter cells that express Fc receptors.

Overall, there is strong evidence that HCV can bind and enter various cell types found outside the liver, and the process of infection can be studied *in vitro *[23]. B cells may preferentially bind free virus, while monocytes may preferentially bind virus-antibody complexes.

### Detection of extrahepatic HCV replication by PCR

PCR is a very sensitive technique for detecting small amounts of nucleic acids. For detecting HCV, the viral RNA is converted to DNA by Reverse transcriptase (RT), and then nested PCR is usually used to amplify particular regions of the genome. The two most commonly amplified regions are the 5'UTR and the hypervariable region 1 (HVR1). The 5'UTR is a highly conserved region that is involved in viral replication and translation [5], while the HVR1 codes for the amino end of the envelope protein E2. E2 is involved in binding of HCV to host cells [24]. Replication of HCV involves converting the viral genomic positive strand into an antigenomic negative strand, and then back to the genomic strand. Thus, the presence of the negative strand strongly suggests that replication is occurring.

We have only analyzed studies that reported RT-PCR of negative strands of HCV in humans. Many other studies have investigated positive strands, but the presence of positive strands is not an evidence of replication unless other data is presented that can only be found in cells replicating HCV. Some of those studies will be discussed in later sections. A number of studies have used model systems such as Chimpanzees and mice for analysis of HCV replication. We have not analyzed these studies, as they are model systems that have not proven useful for studying extrahepatic replication of HCV in humans.

#### Initial results with PCR

The initial attempt to measure HCV replication in sera and livers of patients chronically infected with HCV found negative strands in most of the serum and liver samples [25]. Others followed by investigating the types of infected cells in serum. PBMC was found to contain negative strand HCV RNA by many investigators (Table 1), causing others to investigate infection of particular cell types in PBMC as well as various other tissues or cell types. In the blood, monocytes/macrophages, B cells, and, in one case, T cells were all found to contain negative strands of HCV. Bone marrow cells (BMC) and oral tissues were also found to contain negative strands. For liver [25,26] and serum [25] the ratio of positive to negative strands were estimated to be between 1 and 1000 using semi-quantitative PCR.

**Table 1:** HCV negative strand RNA detection by regular RT-PCR in chronic HCV-infected individuals

Patients, Special groups	Results for negative strand by PCR	Ratio of +/- strands	References
HCC	5/5 L, 0/5 S	1 to 100 L	[26]
Hemophilia	14/27 P, 20/27 PBMC		[148]
Hemophilia	14/19 S, 14/14 PBMC		[149]
HIV-HCV co-infected	4/6 PBMC, 0/6 P	8-50 PBMC	[150]
IFN α-treated	3/11 P untreated; 1/11 treated PBMC		[151]
IFN α-treated	10/10 untreated; 7/9 nonresponders, 2/6 responders PBMC		[152]
Mixed cryoglobulinemia	4/6 PBMC, 5/7 BM		[153]
NHL	6/8 B		[154]
Normal livers	4/4 L, 4/4 PBMC		[155]
Oral lichen planus, oral cancer	3/19 Oral lichen planus; 5/7 Oral cancer		[74]
Chronic	0/5 S, 1/5 PBMC		[156]
Chronic	7/13 PBMC untreated, 5/8 PBMC treated		[157]
Chronic	15/15 L, 0/13 P, 2/3 PBMC, 5/9 BM		[158]
Chronic	0/3 S, 3/3 PBML		[159]
Chronic	L, PBML, B positive (no numbers); Not T, NK		[160]
Chronic	8/9 L, 5/9 S	10-1000 L; 1-10 S	[25]
Chronic	0/19 PBMC, M, T, NK; 3/19 B, 3/19 DC		[73]
Chronic	1/1 L, 10/11 S		[161]
Chronic	28/51 B, 0/14 T, 1/24 M, 19/47 PML		[162]
Chronic	3/5 S, 5/5 PBMC, 3/3 B, 3/3 T, 2/3 M		[163]

Key: L = Liver; S = Serum; P = Plasma; B = B cells; T = T cells; M = Monocytes/Macrophages; BM = Bone marrow cells; DC = Dendritic cells; PBML = Peripheral blood mononuclear lymphocytes; PMNG = Polymorphonuclear granulocytes; PML = Polymorphonuclear leukocytes;

#### Problems with regular PCR

In 1994, the possibility of false positive results for amplifying the negative strand was suggested [27]. It is probably due to self-priming of the RNA or priming by fragments of RNA or DNA present in the reaction. Various improvements to the RT-PCR were tested, such as extended inactivation of RT using heat or Proteinase K, RNase H removal of RNA, and dilution of RT. These modifications reduced the chance of false positives. The use of tagged primers for the RT step also reduced the incidence of false positives [28]. Tagged primers have additional sequence on the 5'end that is not related to the entity being amplified. Another modification to reduce false positives is the use of rTth polymerase [28]. This enzyme works at a higher temperature (70-74 °C), and hence produced a significant reduction in false positives. Therefore, it should be noted that the PCR results described above that used standard protocols may have had false positives.

#### Improved PCR methods for the detection of HCV replication

After 1994, most researchers have followed the recommendations on how to improve RT-PCR to reduce the chance of false positives when measuring the presence of negative strands of HCV. Table 2 shows the results of 23 studies that used one or more of the improvements described above. Again, almost all studies reported finding negative strands of HCV in total PBMC as well as B cells, T cells, monocytes/macrophages, and other cell types.

**Table 2:** HCV negative strand RNA detection by corrected RT-PCR in chronic HCV-infected individuals

Patients, Special groups	Corrections to regular PCR	Results for negative strand by PCR	Ratio of +/- strands	References
Acute, chronic	RNase A	Chronic: 6/12 PBMC; Incubation and acute 0 PBMC (no + or - strands)		[164]
Acute, chronic, IFN α-treated	Heat for 30 min, Core	14/35 chronic PBMC, 1/19 acute PBMC		[165]
Different genotypes	Core	3/11 PBMC, 3/10 CD15+ granulocytes;		[166]
		1/10 CD14+ M, 2/9 CD19+ B, 0/7 CD3+ T		
IFN α-treated	Heat 60 min	Untreated 0/18 S, 13/15 PBML; treated 0/7 S, 3/3 PBML		[167]
IFN α-treated	Heat 30 min	Untreated: 16/17 L, 5/14 S, 7/13 PBMC;		[168]
		Treated, Nonresponders 9/10 L, 3/15 S, 3/7 PBMC		
LT	Heat 1 hr, Core, 3 RT tested	16/20 L, 0/32 S, 2/26 PBMC, 0/6 BM	10 to 100 PBMC	[96]
LT	Core and tag	5/5 L	3 to 340 L	[95]
Mothers	Heat 60 min, RNase, low RT	5/13 PBMC that transmitted to child; 0/13 that didn't transmit HCV		[30]
Occult	Heat 30 min	2/5 PBMC		[169]
Peripheral neuropathy	Tag	0/9 muscle, 0/3 nerve		[170]
Chronic	Chemically modify 3' of RNA	9/10 L, 5/10 S, 1/10 PBMC		[171]
Chronic	Heat 2 hr, RNase H	0/106 P; 33/83 PBMC (40%)		[172]
Chronic	Heat 98 2 hr	0/7 P, 3/7 PBMC	1 to 100 PBMC	[173]
Chronic	Core, RNase, Heat > 20 min	4/8 PBMC		[174]
Chronic	Core and tag	8/10 L, 0/65 S or P, 1/30 PBMC, 0/12 LT PBMC		[175]
Chronic	Tag	L 2/2, 1/6 P, 1/6 DC, 0/6 CD4+, CD8+, CD14+ M, CD19+ B	300-600 L	[176]
Chronic	Tag	29/29 S, 11/29 PBMC, 6/29 PMNG		[177]
Chronic	Tag	3/3 L, 0/3 S	100 L	[178]
Chronic	Heat 45 min	43/45 L, 0/45 S, 37/45 PBMC		[179]
Chronic	Tag, RNase A, RNase H	1/1 L	60 L	[180]
Chronic	NS5	Chronic: 8/8 UTr, 1/3 Tr PBMC; Resolved: 1/10 UTr, 1/1 Tr PBMC;	up to 1000	[58]
		Chronic: 6/8 UTr, 3/4 Tr CD4+ T; 6/8 UTr, 1/7 Tr CD8+ T;		
		Resolved: 6/10 Tr CD4+ T; 1/3 UTr, 2/4 Tr CD8+ T;		
		Chronic: 3/4 UTr B; 3/4 UTr M; Resolved: 4/5 UTr B; 2/3 UTr M		
Chronic	Heat 30 min	13/33 S		[16]
Chronic	Heat 30 min	9/9 L, 0/9 S, 0/9 PBMC		[181]
				

Key: L = Liver; S = Serum; P = Plasma; B = B cells; T = T cells; M = Monocytes/Macrophages; Tr = Treated; UTr = Untreated; LT = Liver transplant; PBML = Peripheral blood mononuclear lymphocytes; PMNG = Polymorphonuclear granulocytes; PML = Polymorphonuclear leukocytes;

The best method for detecting negative strands of HCV uses the enzyme rTth DNA polymerase. Table 3 shows results from 30 studies using rTth to analyze negative strands in various tissues and cell types. HCV negative strands were again usually found in PBMC, as well as in B cells, T cells, macrophages/monocytes, BMC, and other cell types and tissues.

**Table 3:** HCV negative strand RNA detection by rTth RT-PCR in chronic HCV-infected individuals

Patients, special group	Results for negative strand by PCR	Ratio of +/- strands	References
Cadavers	0/6 S, 1/4 lymph; 3/6 CNS	10 CNS to 100 S	[84]
Co-infected, cadavers	7/8 L, 5/8 lymph, 5/8 pancreas, 2/8 adrenal & thyroid, 1/8 BM & spleen	10-100 Various	[29]
Co-infected	15/20 PBMC		[45]
Co-infected	0/10 S, 6/10 PBMC, 4/10 M, 2/10 CD4+ & CD8+ T cells, 1/10 CD19+ B, 2/6 M cultured	10-100 Various	[70]
Co-infected women	13/47 S, 17/48 PBMC		[104]
Co-infected women	61/144 PBMC; 78/315 total specimens		[182]
HCV recovered	9 of 12 PBMC (mitogen stimulated in vitro)		[32]
Humans, Chimps	2/2 S, 0/10 PBMC	10 L; >= 1000 S	[183]
Ischemic heart disease	8/10 Carotid plaque		[184]
Lichen planus	3/3 Skin		[185]
Lichen planus/MC+Cutaneous Vasculitis	0/19 Skin		[186]
LT	42/44, as soon as 7 days post-LT		[94]
LT	0/9 PBMC before transplantation and 3/9 PBMC after		[97]
Lymphoproliferative	4/16 B		[187]
Mixed cryoglobulinemia	6/46 PBMC, 5/46 BM		[188]
Occult	10/10 L, 5/6 PBMC	1-40 L, 1-2 PBMC	[189]
Occult	56/62 L of HCV+ positive, 27/44 L of HCV+ negative		[33]
Occult	11/18 PBMC	10-100 PBMC	[36]
SVR	15/19 L; 12/13 PBMC	6 PBMC, 2.6 L	[57]
Chronic	5/30 in B, 2/30 non-B cells		[190]
Chronic	4 of 17 Oral		[191]
Chronic	6/6 L, 0/6 S	10-100 L	[89]
Chronic	3/3 L	100-1000 L	[192]
Chronic	0/47 Leukocyte, 3/3 Liver	41 L	[193]
Chronic	2/2 L, HCV-strand declines in concentration with increasing time before storage		[194]
Chronic	57/61 L	10-1000 L	[195]
Chronic	5'UTR, Heat 60 min, RNase: 7/9 PBMC, 0/9 S; rTth: 2/3 L, 0/4 S, 0/4 PBMC		[196]
Chronic	4/4 liver, 4/8 PBMC		[197]
Chronic	0/33 P, 0/33 PBMC		[67]
Chronic	39/48 L	10 L	[198]

Key: L = Liver; S = Serum; P = Plasma; B = B cells; T = T cells; M = Monocytes/Macrophages; LT = Liver transplant

We have quantified the number of studies reporting HCV replication in a variety of ways (Table 4). First, we determined the number of studies that found at least one extrahepatic site containing negative strands. Second, we counted the numbers of studies that found any site, including livers, containing negative strands. Third, we determined how many results from particular tissues contained negative strands. Lastly, we determined how many extrahepatic sites contained negative strands. For the last two, we counted each tissue or cell type tested in each study. Unexpectedly, all three categories of PCR methods appear to give about the same results, and all agree that negative strands of HCV are found in extrahepatic tissues.

**Table 4:** Analysis of RT-PCR studies that detected HCV negative strand RNA.

		Studies		
Cell types or tissue	Regular PCR	Corrected PCR	rTth PCR	Totals
L = liver	6/6	13/13	13/13	32/32
S = serum	3/6	6/11	3/6	12/23
P = plasma	2/4	1/3	0/1	3/8
PBMC	10/11	14/16	12/15	36/42
PBML	3/3	2/2	0/1	5/6
B = B cells/lymphocytes	5/5	3/4	3/3	11/12
T = T cells/lymphocytes	1/4	7/11	2/2	10/17
M = Monocytes/Macrophages	1/2	3/4	2/2	6/8
DC = Dendritic cells	1/1	1/1		2/2
BM = Bone marrow	2/2	0/1	2/2	4/5
Granulocytes, PMNG		2/2		2/2
Oral tissues	3/3		1/1	4/4
Carotid plaque			1/1	1/1
Skin			1/2	1/2
Lymph nodes			2/2	2/2
Pancreas			1/1	1/1
Spleen			1/1	1/1
Thyroid			1/1	1/1
Adrenal			1/1	1/1
CNS, brain, nerve		0/1	1/1	1/2
Muscle		0/1		0/1
NK	0/2			0/2

				
**Total for all tissues**	**37/47**	**53/72**	**47/56**	**137/175**
**Percent**	78.7	73.6	83.9	78.3
**Total extrahepatic tissues**	**31/41**	**40/59**	**34/43**	**105/143**
**Percent**	75.6	67.8	79.1	73.4
				
**Studies with > = 1 positive result**	**21/21**	**22/23**	**28/30**	**71/75**
**Studies with > = 1 positive extrahepatic result**	**20/21**	**18/21**	**19/25**	**57/67**

Data from Tables 1 through 3 have been compiled for numeric comparisons between the three versions of RT-PCR as well as overall results for each tissue or cell type.

#### Estimated ratios of positive to negative HCV strands

A number of studies have measured the ratio of positive to negative strands of HCV (Tables 1 to 3). The numbers were determined for liver cells, serum, PBMC, and other cell types. The values range between 1 and 1000, with most studies determining the ratio to be from 10 to 100. Most of these studies were performed before real time PCR became common, so they used semi-quantitative methods that involved diluting samples in 10-fold increments until no band could be detected. Ten of the 22 studies only measured the ratio in livers, four compared liver and other tissues, and the other eight measured only extrahepatic tissues. These numbers do not seem to vary with the RT-PCR method.

#### Conclusions from PCR results

Analyses of the results from RT-PCR studies show that 70% or more of the cell types or tissues that were investigated were positive for negative strands of HCV (Table 4). In other words, HCV replicates in extrahepatic sites. If most of the studies showed extrahepatic replication, why has it not been accepted? One reason may be that the level of negative strands in cells may be too low to be considered a reliable detection. As described above, studies have estimated that the ratio of positive to negative HCV RNA strands is between 10 to100. In addition, the levels of positive strand in extrahepatic tissues has been estimated to be about one-tenth that of the levels in the liver [25,29]. Therefore, there are probably 100 to 1000 fold higher concentrations of positive strand in livers than negative strand in extrahepatic tissues.

Another source of inconsistent results is the patient populations. Studies that showed extrahepatic negative strands usually did not find them in all patients. However, determining why negative strands could not be consistently found is difficult. One study looked at mother to child transmission of HCV, and found that mothers with detectable negative strands in PBMC were more likely to transmit HCV to their child than those without detectable negative strands [30]. This effect may be due to concentration differences, but may be due to other undetermined factors. A study of HCV-positive intravenous drug users (IDU) showed that they were more likely to have negative strand in PBMC than non-IDU HCV-positive patients [31]. This was most likely due to the state of their immune systems, but could be due to other factors such as time after infection. Both these and other studies suggest that negative strand is found in PBMC at varying levels, thereby affecting likelihood of detection.

Occult HCV infection is the presence of liver disease with undetectable or nearly undetectable HCV levels in patient sera. One study used *in vitro *infection of PBMC to demonstrate low levels of HCV present in infected patients [32], while another study demonstrated the presence of HCV in occult infections by using ultracentrifugation to concentrate HCV [33]. For occult infections, detection of positive strands is difficult and thus detection of negative strands would presumably be even more difficult.

One other problem is that although livers are primarily composed of hepatocytes, they also contain large numbers of Kupffer cells (macrophages), sinusoid cells (endothelial), and other cell types in this portal system. The liver studies using PCR did not distinguish the contribution of different infected cell types to the result. Although liver cells always tested positive for the negative strand of HCV, none of the studies determined which liver cell type, hepatocytes, Kupffer, sinusoidal cells, or other cell types, harbored replicating HCV.

One last factor that may have affected this analysis is publication bias. If researchers did not find extrahepatic replication, they may not have published their results. This would cause an overestimate of the studies that found extrahepatic replication. Although it is likely there was some publication bias, we are unable to determine how much of this occurred. This bias would affect the proportion of studies finding extrahepatic replication but not how many studies found it. As 57 different studies using PCR found negative strands in extrahepatic tissues, even if there was a lot of publication bias, the published studies would still provide very strong evidence for extrahepatic replication.

### Detection of extrahepatic HCV replication by other methods

As RT-PCR has established that HCV negative strands are found in various tissues and cell types, we now examine other evidence that HCV replicates in extrahepatic tissues. In addition, some of the techniques described below have been used to determine the percentages of infected cells and/or intracellular locations of HCV.

#### Detection of HCV RNA using *in situ *hybridization

*In situ *hybridization (ISH) can be used to detect viral nucleic acids, and can often distinguish infected cell types, and it can also determine where in the cell the nucleic acid is located. Although the technique can provide nice data when it works, there are a number of problems with the technique. The biggest problem for detecting HCV replication is the low levels of HCV in tissues, as ISH may be three orders of magnitude less sensitive than RT-PCR [34]. In addition, the detection of negative strands is more difficult due to its lower concentration than positive strands. A second problem with detecting negative strands is that the presence of both positive and negative HCV RNA in cells can cause them to form double strands. This makes it harder to detect HCV, as there are fewer single stranded RNA molecules to detect by this method. This problem can be reduced by denaturing the tissue RNA by heating it to 95°C before adding the probe. Both of these problems will cause an underestimation of the number of infected cells and interfere with determining which cells are infected. In addition, there is also natural variability between patients, stage and manifestations of disease, and tissue types. These and other potential problems may make it difficult to compare results and sort out what is actually happening in particular tissues and cells [35]. We are primarily interested in learning if there are conclusive evidence for the determination of replication in particular cell types or tissues.

ISH has been used to study liver cells for evidence of HCV replication. Fifteen studies listed in Table 5 have investigated this question. Of these, ten looked at which cell types were positive for negative strands. Nine stated that hepatocytes were infected, while nine also mentioned infection of other liver cells. Five that mentioned hepatocytes were infected appeared to assume only hepatocytes were infected, as they didn't mention any other cell types in the liver, infected or not. One study stated that all the positive cells were not hepatocytes, while most suggested that other cell types were infected along with hepatocytes. These studies vary considerably in their methodologies, patient populations, and results. However, it appears likely that both hepatocytes and mononuclear cells harbor replicating HCV in the liver. In most of these studies, hepatocytes were more likely to be positive than other cell types.

**Table 5:** Analysis of HCV infection by *in situ *hybridization in chronic HCV-infected individuals

Patients, special groups	HCV region of genome	Results for negative strand	References
Co-infected	5'UTR-core-E1	42/63 liver; Some only hepatocytes, some (portal) monocytes/sinusoidal/biliary	[55]
End-stage liver disease	5'UTR-core-E1	10/20 perihepatic lymph nodes	[41]
HCC	5'UTR, core, NS4B	4/9 HCC hepatocytes; 7/10 Non-HCC	[199]
HCC	5'UTR	Hepatocytes positive, some mononuclear, no biliary or mesenchymal	[200]
LT	5'UTR	2/11 LT patients, 2/11 PBMC, 3/3 mitogen stimulated PBMC	[40]
MC	5'UTR, core	12/12 hepatocytes	[201]
NHL, MC	5'UTR	Ductal and acinar salivary epithelial cells	[42]
Occult	5'UTR	11/18 PBMC	[36]
Oral lichen planus	5'UTR	14/19 oral epithelia	[44]
Sjogren's syndrome or Chronic sialadenitis	5'UTR	8/8 salivary gland	[43]
	35% of genome used	Hepatocytes not infected-macrophages or lymphocytes; No - strand detected	[202]
SVR	5'UTR	15/19 Liver	[57]
Chronic	core-E1	8 of 8 hepatocytes	[56]
Chronic	core-E1	Quantitative image analysis; hepatocytes, some Kuppfer; +/- ratio 3-20	[147]
Chronic	7 regions	Some hepatocytes positive for negative strands	[203]
Chronic	5'UTR	24/28 liver negative strand; 14/17 PBMC negative strand;	[37]
Chronic	5'UTR	9/20 liver-perinuclear in hepatocytes; mononuclear cells had only positive strands	[204]
Chronic	5'UTR	4 of 11, all HIV co-infected-hepatocytes and mononuclear cells	[205]
Chronic	NS5	4/10 H nuclei; 6/10 H cytoplasm; 4/10 M; 0/10 Bile duct epithelial	[54]
Chronic	5'UTR	FISH, positive strands only: 15-70% of liver cells positive, 0.1 to 4% PBMC positive	[39]
Chronic	5'UTR, core	4/5 liver, 5/20 PBMC, 0/20 BM	[38]
Chronic	5'UTR, core, NS4B	4/10 hepatocytes; 2/4 some lymphocytes	[206]
Chronic	5'UTR	17/19 hepatocytes	[34]

Key: LT = Liver transplant; HCC = hepatocellular carcinoma; MC = mixed cryoglubulinemia; NHL = Non-hodgkin's lymphoma; SVR = sustained viral response.

Studies using ISH also have investigated whether replication occurs in extrahepatic sites. Five studies were analyzed to determine whether negative strands could be found in PBMC (Table 5)[36-40]. In all cases, samples contained negative strands of HCV. Studies also found replicating HCV in perihepatic lymph nodes (PLN) [41], salivary gland ductal and acinar epithelial cells [42,43], and oral epithelial cells [44]. The results, therefore, suggest that PBMC is likely a site of extrahepatic replication. In addition, other tissues are likely sites of HCV replication.

The ISH studies consistently showed HCV replication in livers, PBMC, and other tissues. These results agree with the RT-PCR analyses, but there was no agreement regarding which cell types in livers harbor replicating HCV.

#### Detection of HCV proteins using Immunohistochemistry and immunofluorescence

Techniques for the analysis of HCV replication also included the analysis of HCV proteins. As the NS proteins are not found in the virus, evidence that NS proteins are in cells suggests that replication occurred in those cells. The low concentration of HCV in cells makes it difficult to detect NS proteins. In addition, NS proteins may be released from cells and diffuse in the interstitial space to nearby cells. Lastly, due to the variability of different isolates of HCV, antibodies may react against a particular NS protein in cells from one patient but not with proteins from another patient due to epitope specificity. This usually causes researchers to test a variety of antibodies to find the one they believe gives the best results. Frozen tissues generally give better results than formalin-fixed samples. As for ISH, researchers used a variety of methods and antibodies, making comparisons difficult. We were again interested in consistent results from various tissues or cell types.

Studies on liver cells using immunohistochemistry (IHC) or immunofluorescence (IF) for the detection of NS proteins were evaluated, and eight are listed in Table 6. Studies that investigated which cells contain replicating HCV in livers have yielded inconsistent results. Two studies found only hepatocytes stained positive for HCV NS proteins, although one didn't indicate whether they investigated other cell types. Four studies found both hepatocytes and lymphocytes or monocytes containing HCV NS proteins, one found only macrophages, and another found lymphocytes contained them.

**Table 6:** Detection of HCV proteins in various tissues in chronic HCV-infected individuals

Patients, special groups	Method	Proteins	Evidence for NS proteins	References
Acute/chronic	IF	NS3, NS4	NS4 but not NS3 in hepatocytes	[207]
Acute/chronic, IFN α-treated	IF	NS5	17/20 PBMC positive for NS5, all in cytoplasm	[165]
Co-infected, cadavers	IHC	NS5A	Astrocytes and microglial cells in CNS (macrophages) positive	[47]
Co-infected	SUSHI	NS5A	CD14+CD16++ (DC) and CD14++CD16++ (macrophages); not CD14++CD16- (monocytes)	[45]
Co-infected	IHC	NS3	Liver: NS3 more associated with replication than core;	[55]
End-stage liver disease	IHC	NS3	NS3 stained lymph nodes-probably B cells, but likely T and other cells	[41]
HCC	IHC	Core, E2, NS4, NS5	Hepatocytes positive, some mononuclear	[200]
HCC/Cardiomyolitis	IHC	Core, NS4B	Core, NS4B-mostly in macrophages	[109]
NHL, MC	IHC	Core, NS4	Core salivary parotid epithelial, none for NS4	[42]
MC	IHC	Core, NS4	Both liver and skin positive in some samples were positive	[208]
MC	RIBA (Ortho)	NS4	17/37 Sera positive for anti-NS4 antibodies	[209]
MC	IHC	Core, E2, NS4, NS5	3/12 NS4, 5/12 NS5 kidneys; 2/12, 3/12 capillaries; 2/12, 4/12 blood vessels	[49]
Chronic	IHC	NS3, NS5a	3/4 Liver; 4/10 Intestinal with NS3, 3 also with NS5a	[48]
Chronic	IHC	NS4	Hepatocytes, not monocytes, not endothelial, not bile duct epithelium	[210]
Chronic	IF	Core, NS4	4/11 monocytes and B; 3/11 T cells in BM; 1/11 T in PBMC	[46]
Chronic	IHC	NS4	6/14 livers; hepatocytes stained, no mention of non-hepatocytes	[206]
Chronic	IHC	NS4	NS4 mainly in hepatocytes, some Kupffer, bile duct, mononuclear	[211]
Chronic	IF	E2, NS3, NS4 NS5	NS3: mostly CD8+ T, CD20+ B, some CD4+ T. No CD14+ cells found	[108]
Chronic	Confocal	NS5	CD4+, CD8+, B, and M all stained positive (0.02-0.1%) for NS5	[58]
Chronic	IHC	Core, NS4	9/12 for both Core and NS4 in hepatocytes and some monocytes	[201]

Key: HCC = hepatocellular carcinoma; MC = mixed cryoglubulinemia; NHL = Non-hodgkin's lymphoma. IF = immunofluorescence; IHC = immunohistochemistry.

There were also IHC or IF studies that investigated the replication of HCV in tissues besides the liver. Five studies on PBMC are listed in Table 6, as well as another that looked at PBMC constituents. As seen in the table, studies using anti-NS4B, anti-NS5, and two using anti-NS4 all showed positive cells in PBMC. An additional study using a type of flow cytometry called SUSHI also showed positive results for PBMC [45]. They further separated the cells into subsets based on CD14 and CD16 expression and found DCs and macrophages stained positive by anti-NS5A, while monocytes did not. One study found monocytes, B cells, and T cells all infected in PBMC, while T cells in BMC were also infected [46]. The significance of all these studies is they agree that PBMC contains replicating HCV. A study of brain cells using IF found NS5A stained astrocytes and microglial cells (macrophages) [47]. Two of the other studies listed above also found macrophages staining positive for NS proteins. Studies have also investigated replication in other cell types. These found NS proteins in B cells and other cell types found in lymph nodes [41], and others found intestinal epithelial cells [48] and kidney cells [49] stained positive for NS proteins.

The IF and IHC studies consistently showed that extrahepatic sites contain replicating HCV, including macrophages, B cells, and T cells. Studies of liver infections are inconclusive regarding which cell types primarily contain replicating HCV.

#### Other methods

A variety of other methods have been used to investigate the infection of various tissues and cell types by HCV. Although they don't make definitive statements about which cells are infected by HCV, they broaden the range of evidence of replication.

PBMC have been investigated as a possible site of HCV replication by *in situ *RT-PCR [50]. In this technique, cells were isolated, fixed with formaldehyde, and permeabilized. RT is performed, and then tagged PCR is performed using fluorescent primers. They found 12 of 28 PBMC samples positive for negative strand HCV, with between 0.2 and 8% of the cells testing positive.

Two studies have looked at the kinetics of viral infection after liver transplantation. The first investigated levels of HCV after liver transplantation, noting that serum levels of HCV declined for two days, and then increased [51]. They suggested that viral half life was short, and extrahepatic replication contributed little to the level of total virus in the serum. This observation was based primarily on the decline of HCV concentration for two days. However, they did not consider that the repopulation of the liver by HCV infected cells could reduce HCV in the serum, thereby affecting their conclusions. The second group of researchers noticed that the kinetics of HCV concentration in the serum usually exhibited a biphasic curve [52]. This suggested that there were two viral compartments, each with different replication kinetics. In other words, the kinetics suggested extrahepatic replication of HCV occurs.

One study investigated the kinetics of disappearance of HCV RNA from serum and PBMC after pegylated interferon and ribavirin therapy [53]. They found comparable decreases of HCV RNA in both plasma and PBMC, but in some patients the times of decay in PBMC were significantly different than in plasma, suggesting there are at least two cell types involved in replication.

#### Percentages of cells infected *in vivo*

IHC, IF, and *in situ *PCR have all been used to estimate the percentage of cells that are infected by HCV. In our case, we were interested in the percentage of cells that contained replicating HCV. However, only a few papers have determined these percentages.

ISH of liver cells suggested that 1 to 5% of cells were positive, including hepatocytes, mononuclear cells, sinusoidal, and biliary epithelial cells [54]. One patient had as much as 20% of the cells positive. Other studies of liver cells found the percentage of infected cells to be 10 to 50% [55], 14% [56], or 3% [57].

A study of PBMC and BMC by IF produced an estimate that 0.15 to 1% of cells were HCV-infected [46]. In some samples, up to 4% of cells tested positive by anti-NS4. Another study of PBMC by ISH measured the number of infected cells at 3.3% [36]. A study that investigated infection of different cell types in occult infections measured the percentage of cells infected in PBMC by confocal microscopy and found 0.02 to 1% of the cells infected [58].

These studies show that only a few percent of cells are infected by HCV. This number varies widely with study design and cell type, so a more precise number is currently not available. Occult infections show a much lower rate of infected cells.

#### Different cell types could contain different HCV variants

Quasispecies analysis of HCV has been performed by single-strand conformational polymorphism analysis (SSCP) and by sequencing variants that have been cloned. Comparisons between various tissues or cell types were then performed. The 5'UTR and HVR1 are the two areas most often examined, although the NS5B and HVR2 regions have also been analyzed. For SSCP analysis, researchers usually sequence bands that vary between tissues. As one band can harbor more than one variant, these analyses therefore usually underestimate the variation in samples [59]. It is not clear, however, whether this problem would alter the overall conclusions of the studies described below as to whether different tissues harbor different major variants.

Several studies have investigated whether liver variants are the same as other tissues (Table 7a and 7b). Analysis of the HVR1 of HCV found in liver, plasma, and PBMC by one study found significant quasispecies differences between the three tissues [60]. Variants were found in each of the tissues that weren't in the other two tissues. We analyzed eight other studies that determined variants between liver, serum or plasma, PBMC, and sometimes also the PLN. All but one of the studies found differences between the liver, serum, and PBMC, which found small differences in the 5'UTR for some of the patients [61]. In addition, two studies found different HCV genotypes in some patients between liver, serum or plasma, and PBMC [62,63]. These studies show that each of these tissues contain different variants.

**Table 7:** Quasispecies analyses of HCV in various tissues in chronic HCV-infected individuals

Patients, special groups	Cell types investigated	Regions studied	Methods	References
**a. Liver, PBMC, other**				
End-stage liver disease	L, S, PBMC, PLN	HVR1	Seq	[41]
LT	L, S, PBMC, PLN	HVR1	HMA, Seq	[101]
LT	L, S, PBMC, PLN	HVR1	Seq	[66]
IFN α-treated and SVR occult	L, S, PBMC	5'UTR	Seq	[61]
IFN α-treated and SVR occult	L, S, PBMC, PLN	HVR1	Seq	[100]
Chronic	L, P, PBMC	HVR1	Seq	[60]
Chronic	L, S, PBMC	HVR1	Clonal frequency analysis, Seq	[212]
Chronic	L, S, PBMC	HVR1	Seq	[196]
Chronic	L, S, P, PBMC, monocytic DC	5'UTR, HVR1	Seq	[78]
Chronic	L, P, PBMC	5'UTR	INNO-LiPA	[62]
				
**b. Liver, other**				
Cadavers	L, S, CNS, lymph	5'UTR, HVR1	Seq	[79]
Co-infected	L, P, CNS	5'UTR, E1	Seq	[80]
Chronic	L, S	5'UTR, E2-NS2	Seq	[86]
Chronic	L, S	E2-NS2	Seq	[87]
Chronic	L, S	E2-NS2	Seq	[88]
Chronic	L, P, CNS	5'UTR, E1	Seq	[81]
Chronic	L, S	5'UTR	SSCP, Seq	[89]
Chronic	L, PBMC, monocytic DC	5'UTR	Seq	[77]
				
**c. PBMC, other extrahepatic**				
Co-infected	P, PBMC, CSF	5'UTR	Seq, INNO-LiPA	[75]
Co-infected	S, PBMC, M, B, CD4+ T, CD8+ T	5'UTR	SSCP, Seq	[70]
Co-infected	S, PBMC	HVR1	Seq	[105]
Co-infected women	P, PBMC, Genital-cervicovaginal lavage fluid (CVL)	5'UTR	SSCP, Seq	[90]
LT	S, PBMC	5'UTR	SSCP, Seq	[98]
LT	P, PBMC	HVR1	Seq	[99]
LT	S, PBMC	5'UTR	SSCP, Seq	[213]
LT	S, PBMC	NS5B	Seq	[66]
MC	P, PBMC, cryoprecipitate	5'UTR, HVR1	Seq	[65]
Chronic	P, PBMC	5'UTR, NS5B	Seq	[214]
Chronic	P, PBMC	5'UTR, HVR1	Seq	[69]
Chronic	P, PBMC	5'UTR, NS5B	SSCP, Seq, INNO-LiPA	[63]
Chronic	S, PBMC, CSF	5'UTR	SSCP, Seq	[82]
Chronic	P, PBMC	5'UTR	SSCP, Seq	[67]
Chronic	S, PBMC	HVR1,2+	Seq	[64]
				
**d. Other extrahepatic**				
Cadavers	S, CNS (brain autopsies)	5'UTR	SSCP, Seq	[84]
Co-infected	S, CSF	5'UTR, HVR1	SSCP	[83]
Co-infected women	P, Genital-cervical cytobrush	5'UTR, HVR1	Seq	[91]
Co-infected men	P, Semen	HVR1	Seq	[92]
LT	S, Brain	5'UTR	SSCP, Seq	[85]
Oral lichen planus, oral cancer	Oral lichen planus, oral cancer	5'UTR, HVR1	Seq	[74]
Chronic	S, peripheral blood DC	HVR1	Seq	[72]
Chronic	P, BMC	5'UTR	SSCP, Seq, INNO-LiPA	[76]
Chronic	P, B, M, T	HVR1	Seq	[71]
Chronic	P, peripheral blood DC	HVR1	Seq	[73]
Chronic	P, M, B, CD4+ T, CD8+ T	5'UTR	Seq	[58]

Key: L = Liver; S = Serum; P = Plasma; B = B cells; T = T cells; M = Monocytes/Macrophages; DC = Dendritic cells; CSF = cerebral spinal fluid;

PLN = perihepatic lymph nodes; LT = Liver transplant; MC = mixed cryoglubulinemia; SSCP = single-strand conformational polymorphism; HMA = heteroduplex mobility;

We also evaluated 15 studies that analyzed the differences between serum or plasma, PBMC, and other tissues by sequencing and, sometimes, SSCP (Table 7c). These studies did not include liver variants. In all cases compartmentalization between serum or plasma and PBMC was seen in many or all of the patients analyzed. Studies noted lower variability in PBMC than in sera [64,65], that the compartmentalization was stable for over two years [63], and that for liver transplant recipients, the more variable of the donor or recipient strains later became the dominant strain in the transplanted liver [66]. Others noted patients with different genotypes in PBMC and plasma [67,68].

Various combinations of tissues have been examined by a number of researchers (Table 7d). Studies showed that various extrahepatic tissues each had variants that were more alike than variants in the other cell types, suggesting that each cell type has a pool of HCV that was a little different than the pool in the other cell types [58,69-74]. Three of the studies noted that different compartments at times contained different genotypes, as measured by the INNO-LiPA line probe assay. The assay uses HCV RT-PCR products to hybridize to immobilized probes specific for particular genotypes of HCV [63,67,75,76].

If tissues contain different variants, do these differences matter? Two studies analyzed 5'UTR variants in liver, PBMC, and mDC [77,78]. Both studies found mDC variants differed from liver variants. One of the studies investigated *in vitro *translation efficiency of the variants, and mDC variants showed reduced translation compared to the liver variants. A study that investigated liver and serum variants in lymphocytes and central nervous system tissues found 24-55% of the 5'UTR variants absent from serum, while brain and lymph node variants were more similar than either was to liver or serum variants [79]. To determine if different variants affected replication of HCV, quasispecies from brain, serum, and liver were analyzed for *in vitro *translation efficiency. They found that brain derived variants were translated less efficiently than serum or liver. These studies therefore suggest that the quasispecies differences can affect translation efficiency, and presumably other aspects of HCV replication.

Since HCV infection is associated with dementia, investigators have also examined infection of nervous system tissues. Two studies investigated sequences in liver, plasma, and brain and found minor differences in the 5'UTR and major differences in the E1 region [80,81]. Brain specific sequences were identified. Two other studies looked at cerebral spinal fluid (CSF) and found different variants than serum in some patients [75,82], while a third, using SSCP, found CSF and plasma very similar [83]. Since SSCP isn't as sensitive as sequencing for detecting differences and only three patients were studied, it is unclear whether this study could have detected significant differences between these two tissues. In two related studies, brain tissue was compared to serum sequences by SSCP and sequencing from tissue obtained from cadavers [84,85]. Brain and serum variants were significantly different. These studies suggest that various CNS tissues can harbor variants of HCV not found in liver, plasma, or serum.

The kinetics of HCV infection of tissues has also been investigated. Studies investigating the quasispecies in liver and serum found that some patients had significantly different quasispecies in the two samples, including the consensus sequences [86,87], but that the quasispecies complexity varied considerably over time [88]. Variants seemed to appear first in liver, then spread to serum, suggesting most originated in the liver and not in the serum. However, the study could not answer which cells in the liver produce most of the HCV. One study which compared positive and negative strand liver variants and serum variants by SSCP and sequencing showed that four of the patients' liver negative strand variants and serum variants were very similar, while the other two patients had low titers of virus and unreliable results [89]. As the liver positive and negative strand variants differed significantly, it may be that most of the HCV RNA found in the liver may be inside cells but not replicating. As there is 10 to 100 times as much positive as negative strand in the liver, most of the virus could be in cells that don't productively replicate HCV, while it does replicate in another cell type. Nevertheless, this needs further study.

Cervicovaginal lavage fluid (CVL) has also been found to have variants similar to PBMC or serum in HIV-HCV co-infected women, but not in HCV mono-infected women [90]. This suggests that HIV facilitates HCV infection of this tissue. A second study of HIV-HCV co-infected women compared cervical cytobrush and plasma and found unique variants in each tissue [91]. A similar study of HIV-HCV co-infected men showed the presence of HCV in semen [92]. These results suggest that infection by HIV can affect which tissues harbor HCV.

Overall, we analyzed 44 studies, with 43 showing distinct differences between variants found in two or more tissues in many of the patients examined. Some also showed different genotypes in different tissues. One possibility is that these tissues bind and take in different HCV variants, but the virus doesn't replicate in these tissues or cell types. However, the evidence presented above for HCV replication in the same types of tissues, including PCR, viral kinetics, ISH, and IF, leaves little doubt that HCV replicates in a variety of extrahepatic sites and in various cell types.

### Temporal quasispecies variation during HCV infection

Since different tissues contain different quasispecies, studies have investigated how these populations change over time. A recent study followed quasispecies found in four patients over a span of up to 18 years [93]. They suggested there were four stages of HCV evolution: (1) HCV establishes an infection; (2) incremental evolution of variants within subpopulations; (3) diversification into new subpopulations; and (4) strong negative selection in these subpopulations reduces variation and HCV achieves a stable adaptation to the host. Although the small number of patients in the study was a drawback, the model needs further investigation.

A different method of studying temporal variation is to study the repopulation of livers after transplantation. HCV from the cells in the serum, including monocytes/macrophages and lymphocytes, enter and populate transplanted liver tissue, and free virus in the blood also infects livers. We examined four studies that used RT-PCR to examine HCV infection after liver transplantation. Liver specimens had detectable amounts of negative strands by RT-PCR within 7 days after transplantation [94]. Levels of negative strand in the liver after transplants do not correlate with serum levels of HCV [94,95], and negative strands were more likely to be found in transplanted PBMC than in PBMC from individuals with chronic HCV infection [40,96].

We also examined seven studies that compared quasispecies before and after liver transplantation. These studies compared donor and recipient HCV, and determined which type was present in the livers of transplant recipients at various times after transplantation. Studies of liver re-infection by HCV have examined the donor and recipient strains to analyze this process. One early study showed that initially after one transplantation, both the donor and recipient HCV strains could be found in PBMC, but only the recipient strain was found in serum [97], and, after a week, only the recipient strain was still found in both serum and PBMC. This suggested that one strain can take over the infection in an individual. Another comparison of variants with those found in the new livers showed that, for 3 of the 4 patients, liver variants were most similar to those found in serum [98], while another study compared variants in plasma and PBMC after transplantation and found frequent compartmentalization and a decrease in quasispecies complexity, which suggests a genetic bottleneck [99]. Two studies of liver re-infection compared donor and recipient HCV strains over time to show that one strain can overtake or exclude another [66,100], with the more variable strain at the time of transplant overtaking the other strain. A study by another group reached the same conclusions: the most variable strain(s) at the time of transplant was the one that was present much later [101]. Overall, the data suggests that newly transplanted livers are infected by free virus or cells from the blood. The strain of HCV with the most variants is more likely to be able to adapt to the new liver and outcompetes the other strain. How one strain out competes another is unknown.

### *In vitro *translation studies

The IRES of HCV, in the 5'UTR, can be used to measure *in vitro *translation efficiency by using a bicistronic reporter vector. The vector can then be used in an *in vitro *system or inserted into cells using transfection. Luciferase is produced by translation of the reporter vector, so increased luminescence results from increased translation of the reporter gene.

One study analyzed *in vitro *translation of HCV variants found in serum [102]. The variants had different efficiencies for *in vitro *translation and also after transfection into Vero (monkey kidney), HepG2 (liver carcinoma), and Jurkat (T cells). Some of the variants translated better in HepG2 and Vero cells, others in Jurkat cells, suggesting that some variants were better adapted to particular cell types.

As B cells have frequently been found to be infected by HCV, one group investigated the *in vitro *translation efficiency of variants found in B cells and compared them to variants found in plasma but not B cells [103]. The IRES of HCV variants were tested by bicistronic dual luciferase expression in Daudi, Raji (B cells), Huh7 (hepatoma), and primary hepatocytes (PCH). They found plasma IRES were more efficient in Huh7 and PCH, while the B cell IRES were equally efficient as the plasma IRES in Raji and Daudi cells.

These studies show that particular variants translate better in different cell types, supporting the hypothesis that HCV replicates extrahepatically.

### Effect of HIV-HCV co-infection on detection of extrahepatic replication of HCV

Studies of HCV replication in HIV-HCV co-infected individuals have been conducted. Although these studies have been detailed above relating to detection of HCV and quasispecies analysis, the effects of the co-infection on HCV replication have not been detailed. Here we examined two issues: (1) Does HCV replication increase or decrease in co-infected individuals? (2) Is extrahepatic replication enhanced in co-infected individuals? Only studies that compared HCV-mono-infected and co-infected individuals were examined.

Some studies have looked at extrahepatic replication in co-infected individuals. Three studies investigated the HCV positive strand load in extrahepatic tissues and found no significant differences in the average viral load in either tissue in HCV-infected and co-infected individuals [83,104] or in the percentage of infected cells [45]. Another study compared HCV-infected and HIV-HCV co-infected individuals using strand-specific ISH, and found no correlations with HCV RNA levels [55]. They did find a correlation of HCV negative-strands in the liver tissue and disease levels such as liver inflammation and fibrosis. An early study of HCV quasispecies using SSCP in CSF of HCV-monoinfected and HIV-HCV co-infected genotype 1 and 2 individuals showed that both contained variants similar to plasma [83], while a study of HVR1 sequences in HCV-monoinfected and co-infected individuals found higher serum genetic differences in co-infected individuals [105]. This could be due to reduced immune pressure on these individuals, or a greater host range of the virus in co-infected individuals. However, a different study found reduced quasispecies heterogeneity in HVR1 as the CD4+ count dropped [106], which disagrees with the previous study. A recent study compared patients that responded to HAART with non-responders and HCV-monoinfected individuals [107]. There were no significant differences between HCV-monoinfected and HIV-HCV co-infected individuals regarding quasispecies heterogeneity, but patients that responded to HAART showed a ten-fold increase in evolutionary rates of change of HCV quasispecies compared to non-responders. They suggested that the increased heterogeneity may be due to an enlarged viral replicative space, i.e., an increase in the number of infectable cells. One last study analyzed cell types for infection in HIV-HCV co-infected individuals. They found HCV negative strands in PBMC, CD4^+ ^T cells, CD8^+ ^T cells, CD19^+ ^B cells, monocytes, and macrophages [70]. Quasispecies analysis of serum and monocytes/macrophages showed differences, suggesting active replication in monocytes/macrophages, but they did not find HCV negative strands in PBMC of HCV mono-infected individuals. Of the nine studies detailed, three found no significant differences in RNA loads between HCV-infected and co-infected individuals, while the others found differences in quasispecies and/or in which cells were infected by HCV. Therefore, there do not appear to be dramatic differences in RNA levels, but co-infection appears to affect quasispecies and which cell types are infected by HCV. In particular, HIV may reduce the number of types of infectable cells available to HCV.

The effect of co-infection on the CNS has also been studied. One study of CSF in co-infected individuals found that of five individuals that had genotype 3a in serum and PBMC, two had only genotype 1b in CSF, while two others had a mix of 3a and 1b [75]. Another study of HCV mono-infected and HIV-HCV co-infected individuals found 6 of 10 co-infected individuals had detectable HCV in the brain while only 1 of 3 HCV mono-infected individuals were positive [80]. Those that had detectable HCV were more likely to have detectable HIV levels, and brain HCV sequences did not match liver or serum. These studies suggest that co-infection may affect infection of the CNS.

Although the amount of HCV replication in co-infected individuals may not be significantly different, most quasispecies analyses showed variants in CSF, genital tracts, and PBMC that are not present in serum or liver. In addition, co-infected individuals are more likely to have detectable HCV in extrahepatic tissues. Overall, it appears that HIV may not affect overall HCV replication but may affect the range of cell types producing detectable HCV. HCV infects macrophages and T cells, which are also infected by HIV. Studies of how these viruses interact in these cell types will help us understand the interactions between these two viruses.

### Conclusions of *in vivo *studies

We have analyzed studies that have used RT-PCR to detect negative strands in over a dozen different extrahepatic sites. These studies provide overwhelming evidence that HCV negative strands are found outside the liver. As negative strands are evidence of HCV replication, by analogy the RT-PCR evidence strongly suggests HCV replication outside the liver.

Studies that used hybridization or antibody binding and detection were used to observe replication in individual cells. These studies, in theory, can determine exactly which cells are infected and contain replicating HCV. Unfortunately, ISH, IF, and IHC give inconsistent results. Studies using liver biopsies haven't consistently found hepatocytes infected, and often find other positive cell types. The sensitivity of these methods is low, and the possibility of both false positives and false negatives are high. The one consistent result from these studies is that only a small percentage of cells are infected by HCV in any particular tissue.

Another method of investigating HCV replication is to sequence and compare HCV found in various tissues or cell types. The analysis presented above shows rather conclusively that the sequences found in different tissues often differ from each other. Although one explanation is that different tissues bind different variants of HCV, the combination of the presence of negative strands of HCV and different sequences in tissues is very strong evidence that HCV replicates in a variety of tissues and cell types.

Many papers on HCV state that the evidence for HCV replication in hepatocytes is overwhelming. Although hepatocytes are definitely infected by HCV, we were unable to find significant evidence for replication in them. All reported studies using RT-PCR found negative strands of HCV in liver (Table 4). However, the liver contains Kupffer cells (macrophages), B cells, T cells, endothelial cells and others in addition to hepatocytes. Studies using ISH, IF, and IHC have had conflicting results regarding which cells in the liver contain replicating HCV. Most studies used cell morphology to identify cell types, while only three studies that we examined used cell markers to identify infected cells. One used flow cytometry to identify DCs and monocytes/macrophages in PBMC as positive for the presence of NS5A [45]. The second used antibodies to cell receptors to determine which cells were infected in livers [108]. Most HCV-infected cells were CD8^+ ^T cells or CD20^+ ^B cells, with few CD4^+ ^T cells. The third study used antibodies to CD68, CD3, and CD20 to stain cells along with antibodies to NS4 [109]. They found CD68 positive cells contained NS4. Researchers should perform studies using double-labeling to determine which cell types in livers contain replicating HCV.

Liver transplants also provide a window into HCV replication. After liver transplantation, the transplanted liver is almost immediately repopulated by HCV. Studies we examined suggested that the variants repopulating the liver are derived originally from the liver that was in the individual. However, studies investigating the order of infection are scant. Which cells are first infected in the liver? Does the reinfection come from cells in the blood or from free virus? Answers to these questions would either confirm or deny the prevailing idea that hepatocytes are the most important cell type for HCV replication.

The significance of extrahepatic replication needs a lot more study. Some studies have suggested that extrahepatic replication occurs, but is of minor significance. Other studies suggest it has roles in a variety of diseases associated with HCV infection, including B cell lymphomas, hepatocellular carcinoma, and other diseases. A number of studies have investigated the presence of negative strands and treatment outcome. One study, for example, suggested that negative strands in PBMC of mothers are correlated with transmission to their offspring, presumably through the presence of lymphocytes in breast milk [30]. This needs confirmation and further study. To fully understand the significance of extrahepatic replication, models must be proposed and *in vitro *studies performed.

## Which cell types are permissive for HCV replication *in vitro*?

Studies analyzing *in vitro *replication can be broken down into two major groups: (1) short term cell culture demonstrating the presence of HCV in particular cell types and for studies of HCV replication; and (2) long term cell culture that has the goal of studying all aspects of HCV. We will look at examples of each type. We can also break down studies by whether the investigators cultured infected cells from individuals, or infected fresh cultured cells with virus from patient serum or plasma. Last, studies of infection of various cell types have been conducted.

There have been numerous reports of *in vitro *systems, particularly using cultured hepatocytes. Most of these haven't proven useful for further study. Therefore, we have chosen to only discuss systems that have been used enough to produce more than one paper, or ones that illustrate methods or infections of extrahepatic cell types.

### Short term culturing

#### HCV infection of mixed cell types such as PBMC

One method of studying HCV replication *in vitro *is to start with a mixture of cell types found in blood. This has been done by using PBMC as the host for HCV. One early study showed that PBMC could sustain an infection by HCV for about 25 days, with a peak of infection around the second or third week [110]. Viral titers were low. Another study infected PBMC stimulated with phytohemagglutinin (PHA) with HCV from patient plasma and cultured for 15 days [111]. RT-PCR analysis showed HCV was present, and quasispecies analysis showed that variants at the end of the culturing were similar to ones found in patient PBMC. This suggested that the culture mimicked conditions in the blood. Another study found that lymphoblastic cells infected with HCV produced three variants in the 5' UTR [112]. *In vitro *translation of these variants showed greater translation in B and T cell lines compared to HCV strain H77, but not in monocytes or granulocytes.

Another method to study HCV replication is to separate the components of PBMC, infect the different cell types, and determine which cells are positive for HCV. One study separated PBMC into CD4^+ ^T cells, CD8^+ ^T cells, and CD19^+ ^B cells [113]. After infecting them and PBMC, after about two weeks B cells and PBMC contained minus strands, suggesting replication.

Some HCV-infected individuals that have been treated have undetectable amounts of HCV in the serum and plasma using standard methods. One method that has been used to detect HCV in these patients is to culture patients' PBMC. In many cases, HCV replicates sufficiently in PBMC to be detectable. One such study investigated hemophilic patients [114]. They found 5 of 6 PBMC cultures produced HCV. HCV-mono-infected individuals had PBMC that produced HCV for about 25 days, while HIV-HCV co-infected individuals had PBMC that produced HCV for around 35 days. They further investigated this phenomenon and found that PBMC cultured from co-infected individuals are more likely to produce HCV than cultures from HCV-mono-infected individuals [115]. In addition, they isolated B cells transformed by Epstein-Barr virus (EBV) which could continuously produce HCV for extended periods of time.

#### HCV infection of lymphoid cells

An early report on HCV infection of T cells used serum from an HCV-infected Chimpanzee to infect Molt-4 cells [116]. Negative strand HCV was seen from 3 to 7 days PI, and intermittently detected for up to three weeks in retroviral infected cells. Infection of cells that were not co-infected was less efficient. Another report of infection of MT-2 T cells found negative strands at 10 days PI and positive strands for up to 15 days [117], and they later selected for variants that could be detected in culture for at least 30 days PI [118]. Testing of the MT-2 cell line as well as an immortal hepatocyte cell line called PH5CH showed that culturing HCV isolated from sera affected the variants in HVR1, and the variants in the two cell lines were different [119]. These results suggested cell tropism of HCV. Analysis of the complete genome of the HCV also showed that the cultured HCV became progressively more homogeneous during the culture period [119].

The first report of *in vitro *infection of monocytes/macrophages was using HCV from HIV-HCV co-infected individuals [70]. The monocytes/macrophages were purified and cultured *in vitro *to determine if they were infectable. Two of seven cultures had negative strands of HCV for three weeks. One of the two samples had different SSCP patterns after culturing than before. This suggested that the cells were selecting particular variants. A following study used fresh macrophages isolated from healthy blood donors for infection by HCV [120]. They found that 15 of 26 sera from HCV-infected individuals produced detectable negative strand HCV in 2 to 3 week cultures. Analysis by SSCP and sequencing of the 5'UTR showed four macrophage cultures had different sequence variants than sera. They also showed that cell supernatants of HCV-infected cultures had more IL-8 and TNF-α than uninfected cultures. A third study by the same group infected fresh macrophages with HCV and/or M-tropic HIV [121]. All cultures infected first by M-tropic HIV and then HCV were HCV positive, while only 3 of the 6 cultures infected only by HCV were positive. In a similar set of experiments for Daudi cells that expressed CD4, all HIV and HCV infected cell cultures were positive for HCV, while 5 of 6 infected only with HCV were positive. HIV therefore facilitates infection by HCV in macrophages, but probably not in CD4^+ ^T cells.

The first *in vitro *HCV infection of fresh monocytes/macrophages was performed using cells isolated from buffy-coats obtained from healthy donors [122]. Negative strands of HCV were usually found for up to 10 days after infection, while positive strands were found for up to 27 days. Quasispecies analysis showed that the complexity of the variants decreased after culturing, and serum and monocytes/macrophages had different major variants. Since monocytes/macrophages isolated from blood could be infected by HCV, a follow-up study could find no cytopathic effects or negative strands in Kupffer cells obtained from liver cancer patients [123].

Daudi cells have been investigated as a host for HCV replication. One study showed that HCV in Daudi cells can be detected for up to 19 days, and the total amount of HCV increased 30-fold [124]. They found that enlarged Daudi cells were positive for HCV by ISH. They also found virus-like particles about 42 nm in size in the cytoplasm of cells displaying early signs of apoptosis. Membrane bound organelles were common, as were intracytoplasmic electron dense inclusion bodies.

Immature DC and mature DC were investigated as a target of HCV [125]. HCV positive strand, were detectable for up to 10 days PI, while negative strands could be seen as early as day 2. In mature DC, the positive strand was detectable for only 5 days PI, and the negative strands on days 1 and 2 PI. They also compared quasispecies in the HVR1 of the inoculums and immature DC positive strands at 6 days PI and found differences, suggesting selection of particular variants by immature DC.

#### Synthetic systems

The first major development of a synthetic model system for studying HCV was a Replicon model [126]. A revised model system was later developed that contained much more of the genome, and is called JFH-1 [127]. The JFH-1 genome is derived from a patient that had fulminant hepatitis, so probably is a particularly virulent strain of HCV. Huh-7.5 hepatoma cells are especially permissive for JFH-1, but attempts to replicate the HCV model in monocytes, macrophages, DCs, B cells, and T cells all failed [128]. This study highlights the problems in using model systems. Even though a synthetic HCV system was developed that works *in vitro *in one particular type of permissive cell, it doesn't appear to mimic HCV *in vivo*. Huh-7 cells are derived from an individual with hepatocellular carcinoma, and have a defective HFE protein [129], which is involved in hemachromatosis, and a defective Fanconi anemia gene, FANCC [130]. Huh7.5 cells, derived from Huh7 cells, are also defective in RIG-I [131]. The model systems have been useful for studying intracellular locations of HCV proteins. They have also been useful for reverse genetics, where mutations are introduced and the effects analyzed. However, studies using synthetic model systems should be interpreted with caution, as the restricted host range and unusual nature of the Replicon genome casts doubt on the applicability of the results.

### Long term culturing

Long term culturing of HCV can be accomplished by isolating already infected cells from HCV-infected patients. One such B cell line was established from a patient that had type II mixed cryoglobulinemia (MC) and non-Hodgkin's B-cell lymphoma (NHL) [132]. The cell line, SB, can produce virus that is able to infect fresh Raji cells (B cells) *in vitro*. Quasispecies analysis of the virus in SB showed it had similar variants as spleen, but were different than serum. They also established two cell lines by using HCV-infected PBMC, and then EBV-immortalized B cells. A follow-up study used virus produced from SB to infect the T cell lines Molt-4 and Jurkat *in vitro *[133]. They could detect positive and negative strands as well as viral proteins. The virus in the T cells could be passaged twice, but not for a third time, suggesting the virus was becoming inactivated. Another study using SB showed that it could infect CD4^+ ^T cells, CD14^+ ^monocytes/macrophages, and CD19^+ ^B cells [134]. As CD4^+ ^cells were the most susceptible to virus produced from SB, they further analyzed this system. Production of interferon-γ was reduced and CD45RA expression declined, suggesting that Th1 development was interrupted, and apoptosis increased.

A bone marrow derived B cell line named TO.FE was found to be susceptible to infection by HCV [135]. HCV could be cultured in these cells for at least 6 months. TO.FE carries endogenous EBV, so it is an immortal cell line. The infected cells could produce virus-like particles in the cytoplasm having a diameter of about 45 nm [136]. They also found cytopathic effects such as enlarged cytoplasmic vesicles containing amorphous particles. The persistently infected TO.FE_HCV _was also used as donors of HCV to HepG2 cells in co-culture [137]. HepG2 are not infectable by free virus, so transfer was likely from cell-to-cell contact. This study suggests that HCV may spread into hepatocytes primarily through cell-to-cell contact instead of infection by free virus. A following study investigated cell-to-cell transmission of HCV from TO.FE_HCV _cells to 2.2.15 hepatoma cells [138]. HCV could still be detected 120 days after infection of the 2.2.15 cells, but infections using free virus could not be detected. Quasispecies analysis of the HCV after 4 months of culture in B cells showed variants not seen in the initial serum used to produce the B cell line. The same group also analyzed infection of the TO.FE cell line and stained infected cells using anti-NS3, anti-NS4, and anti-E2. Stain for these proteins was found in the perinuclear space, the golgi apparatus, and the endoplasmic reticulum [139].

An *in vitro *system using PHA-induced T cells has also been developed [140]. They detected positive strands and negative strands of HCV, as well as the proteins NS5A and E2. Virus could be detected for at least 11 days PI, and passaged performed multiple times in T cells. They later used the system to show that individuals with sustained virological response (SVR) could still infect T cells [141]. The infection of T cells was blocked by anti-E2, anti-CD81, and interferon-α.

**Figure 2:**
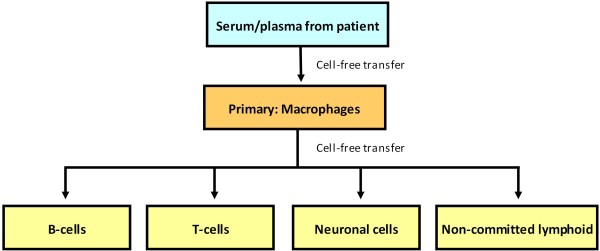
**Flow chart for long term in vitro culturing of HCV isolated from patient serum or plasma**. Figure adapted from reference [23].

**Figure 3:**
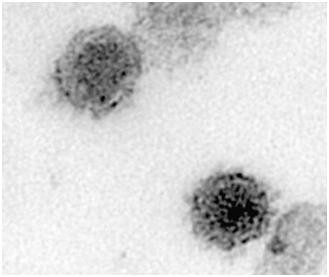
**Electron micrograph of mature (black arrow) and immature (red arrow) HCV from *in vitro *infected T-cell**. Picture adapted from reference [146].

**Figure 4:**
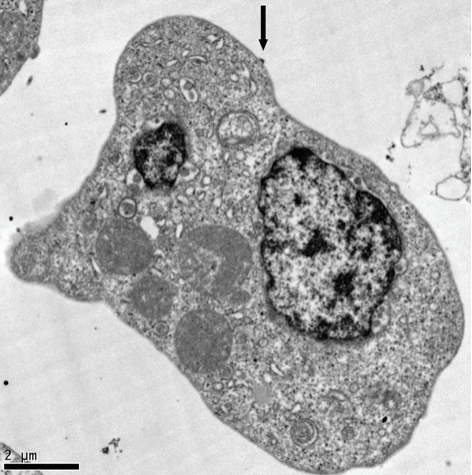
**Example of a triply-infected K7 cell**. TEM showing HIV-1 particle budding from the plasma membrane (arrow). Figure from reference [146].

**Figure 5:**
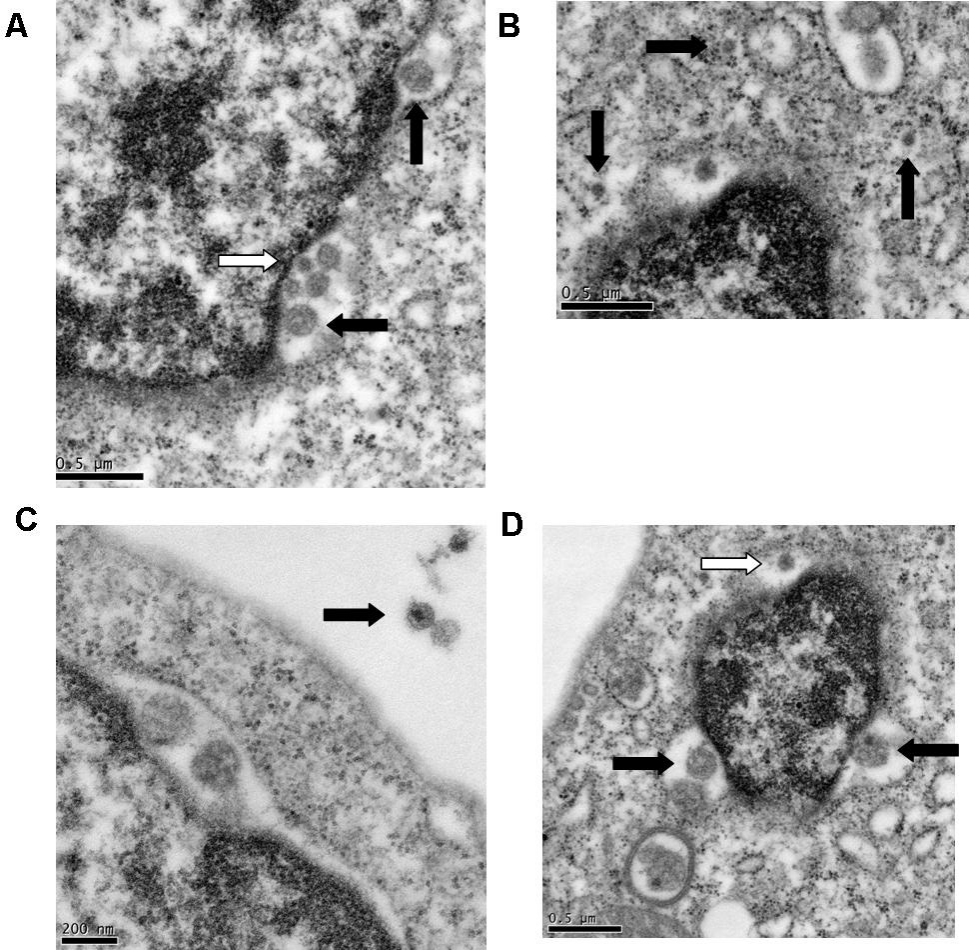
**Detail from triply-infected cell from Figure 4**. (A) HHV-6 shown budding from the nuclear membrane (black arrows) and HCV located in the perinuclear space (white arrow); (B) HCV particles observed in the cytoplasm; (C) HIV-1 particle located outside the cell. (D) HHV-6 (black arrows) and HCV (white arrow) located in the perinuclear space. Figure from reference [146].

Culturing of HCV in PBMC has been performed by multiple groups, but after about 25 days the HCV titers drops. This is probably due to changes as the macrophages start to mature after about two weeks of culture. They change from releasing cytokines that promote other cell types to releasing cytokines that inhibit them [142]. A novel culture system was therefore developed to take into account the properties of PBMC and its components (Figure 2). The system uses HCV from serum or plasma to infect macrophages. The macrophages and other cell types are purified from umbilical cord blood. After a week of culture, a cell-free extract of HCV is used to infect other cell types [23]. The virus is called CIMM-HCV (Figure 3). EBV infected B cells have been used to culture HCV by this system for over two years. T cells, neuronal cells, and non-committed lymphoid cells can also be infected using this system. Cell lines selected for permissiveness to HCV were not used in this system, so the replication of HCV in this system is likely to be closer to natural replication than the other systems described above. In addition, it can be used to study replication in a greater variety of cell types than the other long term culture systems. Analysis of the 5'UTR of HCV cultured using this system showed only minor changes when HCV genotype 1 was cultured in these cell types [143]. Comparison of sequences of the 5'UTR for HCV samples containing deletions or that were genotype 3 provided evidence that cord blood macrophages select for 5'UTR sequences similar to HCV genotype 1 [144,145]. As both macrophages and T cells can be infected by HCV using this system, the system has been used to co-infect T cells with HCV, HIV, and HHV-6 [146]. Cells containing all three viruses were observed (Figures 4 and 5), demonstrating that this isolation system can be used to study co-infection by these three viruses. As noted above, it is unclear how or whether HIV and HCV interact *in vivo*, so this system could provide a method to investigate interactions between these viruses.

### Conclusions of *in vitro *studies

Short term *in vitro *systems have been useful for demonstrating the presence and replication of HCV isolated from particular tissues or cell types. However, studies of the infectious process are best done using *in vitro *systems that can culture HCV for months or years.

We have described above a long term culture system that isolated infected B cells from one patient. This culture, SB, produces virus that has been used for several further studies, and has been used to infect other cell types. The limitation of the system is that there is only one version of the virus. The second culture system found a permissive cell type, TO.FE, which is also able to produce virus for extended periods of time. The virus produced has been studied as well as transferred to hepatoma cells. The limitations of this system include the use of a permissive host and that it is limited to B cells. It is also unclear if it can be used to reproducibly isolate HCV from sera or plasma. Culturing of virus produces variants, and using a cell line that is permissive suggests that it harbors mutations that allow HCV to grow. The third long term culture system can be used to infect T cells with HCV. The limitation of this system is that it only can be used to culture HCV in T cells. It has not been used to study HIV-HCV co-infection. The fourth and last long term culture system does not use cell lines. Instead, the system uses cells purified from cord blood for the culturing. This has the advantage of using normal cells that are not defective in preventing HCV replication, and it can be used to study infection of a variety of cell types. However, the disadvantage is that additional effort is required to isolate the cells used in this system. The system may mimic a more natural infection where HCV passes from one cell type to another. In addition, its ability to promote the infection of a wider range of cell types gives the *in vitro *system a unique advantage: it has been demonstrated that it can be used to study co-infections of HIV, HCV, and/or HHV-6.

## Importance and significance of extrahepatic infection by HCV

Since little is currently known about HCV replication, it is essential that studies be performed to try to understand the biology of the virus. We therefore will discuss some models of how HCV replicates in humans, and suggest how to test these models.

Model 1: HCV replicates in hepatocytes. The infectious virus is passed to other hepatocytes either through production of free virus or by cell-to-cell contact. This model is probably the model favored by most HCV researchers. The model would claim that extrahepatic infection is not important and probably a result of variation of the virus. Another corollary of this model is that HCV does not replicate in extrahepatic tissues.

The evidence presented above refutes the notion that HCV doesn't replicate extrahepatically. However, the main claim is that HCV replicates from hepatocyte to hepatocyte. The lack of culture systems for hepatocytes suggests that this claim is relatively weak. In addition, evidence that HCV replicates only in hepatocytes is poor. For this model to be accepted, there would have to be a preponderance of studies that show that HCV does not replicate in other liver cell types. Studies have suggested that infection of livers can occur as foci, where a clump of cells is infected in one place, another clump in another place, and so on [147]. What cell types are in the clump? To date there are contradictory studies on this. Furthermore, there would have to be a culture system developed that could take HCV from serum or plasma and culture it for extended periods of time. To date neither of these has been accomplished. One other note is that the current synthetic culture system using JFH-1 used sequences from a patient that had fulminant hepatitis. This suggests that the JFH-1 strain was derived from a virus that had adapted to primarily infect hepatocytes, thereby causing fulminant hepatitis. Most patients do not seem to have an HCV strain adapted to only infect hepatocytes.

Model 2: HCV infects macrophages, produces free virus, and transfers it to B cells and hepatocytes. An example for this model is the role of macrophages in harboring large intracellular amounts of HIV. This presumably maintains a reservoir of HIV *in vivo*, enabling virus released later from the cells to infect various cell types. Similarly, the infection of macrophages could be from free virus or from virus-antibody complexes.

The method that HCV uses to infect macrophages or monocytes has not yet been determined. If immune complexes are the method of infection, then the cells should use Fc receptors for binding and entry. As noted above, there has been one study that suggests this method may be used, but it needs to be repeated by others. It may be that free virus also infects macrophages, and HCV could use two different methods to infect macrophages. In addition, the receptors for other cell types also need to be determined. How does HCV infect B cells? How does it infect T cells? Does the genotype of the virus matter for extrahepatic replication? *In vitro *studies described above suggest that HCV genotype 1 preferentially replicates in macrophages. Would this account for the difficulty of treatment of that genotype?

Model 3: HCV infects lymphoid cells, which pass virus to hepatocytes, which pass virus to lymphoid cells. Studies described above suggest lymphoid cells can be infected by HCV, and can pass the virus to hepatocytes. The passages could therefore be due from free virus or cell-to-cell contact. Additional and more definitive studies of infected cells in the liver would help determine if HCV replicates in lymphoid cells, macrophages, and/or hepatocytes in the organ.

We are sure that other models of HCV infection can be developed and tested. The main issue is that nobody is developing and testing such models.

## Conclusions

Although HCV was identified over 20 years ago, there are many unanswered questions about this virus. The major question is the details of the entry and the replication cycle of HCV. We have presented a large amount of evidence from a variety of labs using a number of different techniques to address a variety of questions, including host range and target specificity in terms of immune reactivity. The *in vivo *studies described above have provided an impressive amount of evidence that HCV infects not only hepatocytes, but many other extrahepatic sites that include B cells, T cells, and macrophages/monocytes. The life cycle of HCV is unclear, but may involve cycling between liver and other tissues. A complex life cycle could help explain why it takes a long time for HCV-infected individuals to progress to liver and other diseases. Although they can be difficult to perform, more *in vivo *studies that address the life cycle of HCV are needed, particularly studies of the infection of livers.

Systems that study the natural virus *in vitro *need to be supported and exploited. The infectious process in macrophages, B cells, T cells, and other cell types need to be studied to determine if the current knowledge of infection and spread of HCV in specially engineered hepatoma cells by synthetic systems is meaningful. Studies using synthetic model systems have serious problems which have been largely ignored. The Replicon studies have avoided and masked the questions related to the real virus. Now that extrahepatic replication has been established, studies should be performed to determine its significance. Even the Replicon related studies should be focused on non-hepatic cells. This new area should be considered essential to understanding the life cycle of HCV, and not an irrelevant adjunct to the current mainstream of attention. Another significant area for investigation is the different genotypes and the hypervariable regions of the HCV genome.

Overall, there is a great need to be a much broader understanding of the biology of HCV in addition to collecting proteins for potential therapy.

## Competing interests

The authors declare that they have no competing interests.

## Authors' contributions

Both authors wrote and approved the final manuscript.
